# A case report of a new pulmonary embolism occurring in a patient receiving continuous infusion of recombinant activated protein C

**DOI:** 10.1186/1749-7922-1-23

**Published:** 2006-08-09

**Authors:** Bernard J Benedetto, Michael A Houston

**Affiliations:** 1 Rhode Island Hospital, Brown University School of Medicine, 2 Dudley Street, Providence, RI, USA; 2 University of North Carolina Medical Center, University of North Carolina School of Medicine, Chapel Hill, NC, USA

## Abstract

**Background:**

There are no guidelines governing the concomitant use of recombinant human activated protein C (rhAPC) and deep venous thrombosis/pulmonary embolism (DVT/PE) prophylaxis in critically ill patients. It is unknown if rhAPC provides any protection against DVT/PE in this population of patients.

**Methods:**

Case report.

**Results:**

This report describes the first case of a radiographically demonstrated pulmonary embolism occurring in a patient receiving continuous therapeutic infusion of rhAPC.

**Conclusion:**

The administration of rhAPC alone may not be sufficient DVT/PE prophylaxis in high risk patients. The risks associated with concomitant anticoagulation and rhAPC therapy are unknown. Further research is necessary to determine the safest and most effective regimen for DVT/PE prophylaxis in patients receiving rhAPC.

## Background

Recombinant human activated protein C (rhAPC) has been demonstrated to improve mortality in critically ill patients with septic shock [1]. It is thought that rhAPC exerts its effect via multiple mechanisms involving anti-inflammatory, antioxidative, antiapoptotic and anticoagulant pathways [2]. The anticoagulant effect of rhAPC leads to a 2–3% incidence of severe bleeding complications in treated patients [1]. This risk has led to the general practice of discontinuing the use of all other forms of anticoagulation, including deep venous thrombosis (DVT) prophylaxis, when rhAPC is used. It is unknown what DVT or pulmonary embolism (PE) prophylaxis is safe and appropriate in patients receiving rhAPC. Further, it is not known if rhAPC alone provides any protection against DVT/PE. We present a case report of a patient who developed a new, documented PE while receiving a continuous infusion of rhAPC.

## Case presentation

**Figure 1:**
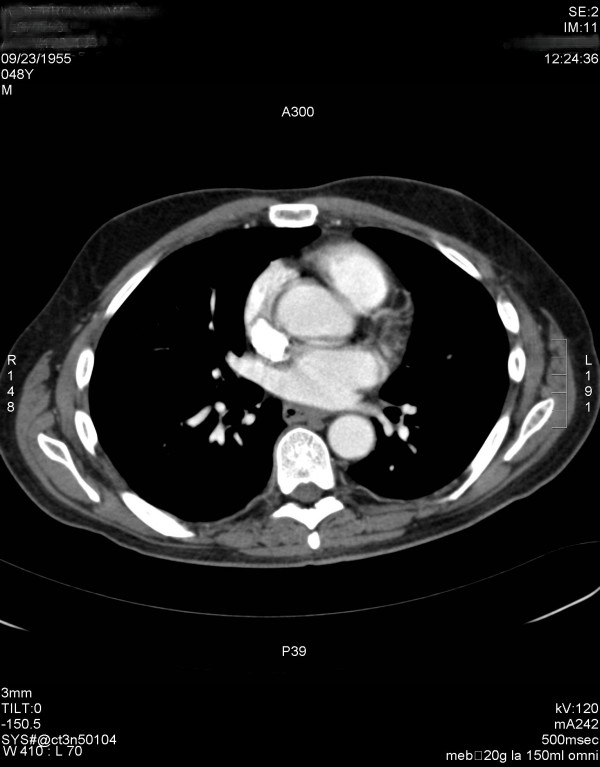
CT Angiogram obtained prior to infusion of rhAPC demonstrating no evidence of pulmonary embolism.

A 48 year old male resident of a psychiatric institution presented to the emergency department with diarrhea, vomiting and decreased mental status. He had a history of hypertension, schizophrenia, hypothyroidism and factor XII deficiency. He was not receiving any anticoagulation for his Factor XII deficiency at the time of presentation. According to his emergency department record he was febrile to 38.7 Celsius with mild abdominal distension and tenderness on physical examination. A computed tomographic (CT) scan of his abdomen did not demonstrate any intraabdominal abnormality. An empiric diagnosis of infectious diarrhea with dehydration was made and the patient was discharged back to his facility on Levofloxacin and Metronidazole and intravenous fluids. Three days later, he represented to the emergency department with persistent diarrhea and a metabolic acidosis. A repeat CT scan was obtained which demonstrated an ill-defined rectosigmoid mass, but no obstruction. The pulmonary artery was seen on the uppermost cuts of this scan and there was no evidence of pulmonary embolus at this time (Figure 1). Sigmoidoscopy revealed no mass or mucosal abnormality and the patient was admitted to the medical intensive care unit with continued broad spectrum antibiotics, intravenous hydration and hemodynamic monitoring. He was hemodynamically stable. Sequential compression devices were documented to be in place for DVT/PE prophylaxis.

On hospital day two, the patient developed increasing abdominal tenderness which was associated with fevers up to 39.5 degrees Celsius and acute renal failure. Surgery was consulted and recommended urgent operative exploration. Upon exploration the patient was found to have mesenteric venous thrombosis with a segment of ischemic small bowel. He underwent small bowel resection and was returned to the intensive care unit for continued resuscitation. At this time a heparin infusion was initiated given the patient's known factor XII deficiency and demonstrated mesenteric venous thrombosis. His partial thromboplastin time (PTT) was maintained between 60 and 80 seconds. The following day he had not improved; he was returned to the operating room where a second segment of ischemic bowel was discovered and further resection was performed. The patient again returned to the intensive care unit in critical condition requiring pressor support with levophed and continued ventilatory support with a PaO2 to FiO2 ratio of 180. At this point he was evaluated and found to be a candidate for rhAPC. This was initiated six hours after the completion of his operation. Due to concerns about potential bleeding complications, the heparin infusion was discontinued when the rhAPC was started. At the time of heparin discontinuation the patients PTT was 82 seconds and he had no clinical evidence of DVT or PE. His activated protein C level at that time was less than 10.

**Figure 2:**
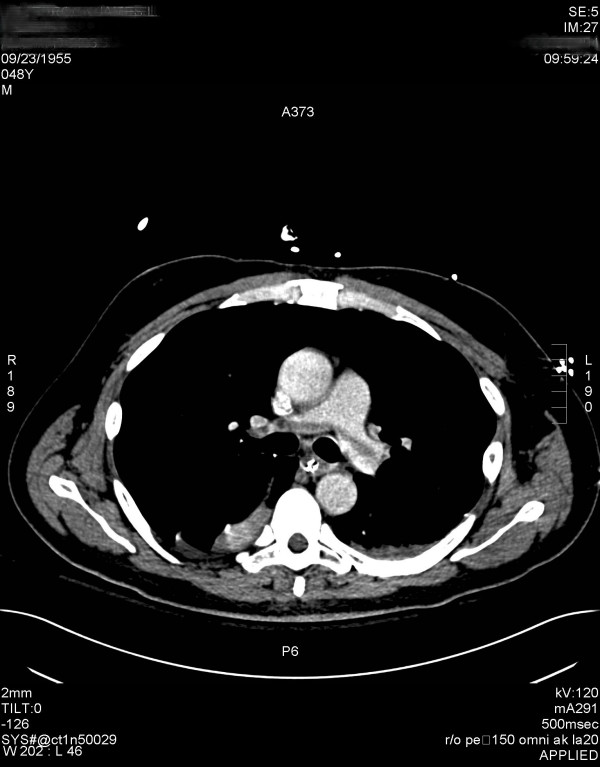
CT Angiogram obtained after 78 hours of infusion of rhAPC demonstrating a large saddle pulmonary embolism.

Over the course of the following two days, the patient showed significant hemodynamic improvement and pressors were discontinued. By day 3 PaO2 to FiO2 ratio had risen to over 300 and he was requiring minimal ventilatory support. On postoperative day 4, the patient developed acute hypoxia with increasing ventilatory requirements. Spiral computed tomographic scan of his chest with intravenous contrast demonstrated a large saddle pulmonary embolism (Figure 2). Heparin infusion was reinstituted and the rhAPC drip was discontinued after 78 hours of the 96 hour course. Doppler studies of the lower extremities and echocardiography revealed no evidence of deep venous or mural thrombus. Subsequently, the patient weaned from the ventilator and was extubated on postoperative day 8. He was transferred to a hospital ward and oral anticoagulation was begun on postoperative day 9. The patient was discharged on postoperative day 13 on oral anticoagulation with an INR or 2.2.

## Conclusion/discussion

Critically ill patients are known to be at high risk for the development of DVT/PE with a rate of thromboembolic complications ranging between 9% and 29% depending on the specific population studied [3-6]. Multiple clinical trials have demonstrated that DVT/PE prophylaxis improves clinical outcomes in the critically ill [7,8]. The Society of Critical Care Medicine guidelines for the treatment of patients with sepsis currently recommends that all critically ill patients receive DVT/PE prophylaxis with either subcutaneous heparin or low dose low molecular weight heparin unless otherwise contraindicated [9].

Whether administration of rhAPC should be a contraindication to the administration of DVT/PE prophylaxis with subcutaneous or low molecular weight heparin is not known. The PROWESS trial (Recombinant Human Activated Protein C Worldwide Evaluation in Severe Sepsis) offers some data on this subject. Subgroup analysis of mortality rates according to exposure to heparin in the PROWESS trial raised the possibility of a heparin/rhAPC drug interaction [10]. The relative risk of 28-day all-cause mortality was increased in the group who received both heparin and rhAPC as compared to those who received only rhAPC. This increased relative risk of mortality was not due to an increase in bleeding complications in the heparin/rhAPC group, raising the possibility that heparin might reduce the efficacy of rhAPC. Subsequent biological data has demonstrated that heparin increases the affinity between native APC and alpha-1-proteinase inhibitor and a plasma protein C inhibitor, both of which decrease native APC activity [11]. The *in-vivo *effect of heparin on rhAPC binding with alpha-1-proteinase inhibitor and plasma protein C inhibitor is not known. Further confounding this issue is the fact that the presence of the heterozygous mutation for the factor V leyden mutation does not seem to impact the effect of rhAPC [12].

Bleeding is the most significant complication associated with rhAPC with a 1–2% incidence of significant bleeding complications. At the doses used for DVT/PE prophylaxis neither subcutaneous nor low dose low molecular weight heparin have been associated with an increased risk of serious bleeding complications. Low dose low molecular weight heparin has, however, been associated with an increased rate of wound hematomas in post surgical patients. It is not known if concomitant administration of rhAPC and either subcutaneous or low dose low molecular weight heparin produces a synergistic increase on the risk of significant bleeding complications.

It is known that the activity of native activated protein C is diminished in critically ill patients [13]. It is also known that patients with congenitally low levels of native protein C activity are prone to both micro and macrovascular thrombosis [14]. Diminished levels of native protein C activity may play a role in the increased risk of DVT/PE demonstrated in critically ill patients. Modulation of this hypercoagulable state has been hypothesized to be the primary mechanism by which rhAPC exerts its beneficial effects. It is also thought to be this pathway that leads to the increased bleeding risk associated with its use. What impact this pathway has on DVT/PE risk is unclear. One animal study has demonstrated that the administration of rhAPC prevents thrombin induced DVT/PE in mice [15]. The ability of rhAPC to prevent the development of DV/PE in this very high risk animal model is encouraging but the clinical relevance of this has not been elucidated.

There are also several other confounding factors that become relevant when dealing with the issue of rhAPC use and concomitant DVT/PE prophylaxis in individual critically ill patients. The kinetics of native protein C activity varies greatly between individual patients [16]. While the activity of native activated protein C has been demonstrated to decrease in some critically ill patients, this decrease is not seen in every patient. Furthermore, even in those patients who do have decreased levels of native activated protein C activity, certain patients have earlier rebound of native protein C activity while others may have prolonged suppression of activity. These variations in individual native activated protein C kinetics could lead to a wide variation of the degree of anticoagulation produced by rhAPC administration. Further, individual abnormalities of the coagulation cascade, whether acquired or genetic, are being identified with increasing frequency in the general population [17]. It follows that these abnormalities occur with a similar frequency in the critically ill patient population as well. These factors, and others, will need to be more clearly defined in order to determine each patient's individual risk of DVT/PE and potential benefit or harm gained from the use of rhAPC.

In conclusion, this is the first known case report of a radiographically documented PE occurring in a patient receiving continuous infusion of rhAPC. This case suggests that rhAPC alone may not be sufficient DVT/PE prophylaxis in high risk patients. The risks associated with concomitant anticoagulation and rhAPC therapy are unknown. Further research is necessary to determine the safest and most effective regimen for DVT/PE prophylaxis in patients receiving rhAPC.

## Competing interests

The author(s) declare that they have no competing interests.

## Authors' contributions

BB conceived of the case report, participated in the chart and literature review and drafted the manuscript. MH participated in the chart and literature review and proofread the manuscript. All authors have read and approved of the final manuscript.
